# A novel scoring system to predict the residual back pain after percutaneous kyphoplasty for osteoporotic vertebral compression fracture

**DOI:** 10.3389/fsurg.2022.1035681

**Published:** 2022-10-13

**Authors:** Dongjun Yang, Xin Liu, Yang Zhou, Yong Xu, Qiangkai Huang

**Affiliations:** Department of Orthopedics, Second People’s Hospital of Chengdu, Chengdu, China

**Keywords:** osteoporotic vertebral compression fracture (OVCF), percutaneous kyphoplasty (PKP), residual back pain, prediction, scoring system

## Abstract

**Objective:**

To establish a scoring system to predict the residual back pain after percutaneous kyphoplasty (PKP) for osteoporotic vertebral compression fracture (OVCF).

**Materials and methods:**

We retrospectively reviewed the clinical records of 98 patients who were diagnosed of single-vertebral OVCF and underwent PKP surgery in our department from January 2015 to December 2017. The following clinical characteristics including age, gender, disease course, fracture location, fracture type, segmental kyphosis, and bone cement volume were all recorded, and the effects of these factors on postoperative pain (at 1-month and 6-month postoperative) were also analyzed respectively. Based on 6-month postoperative VAS score, the included patients were divided into two groups, namely the residual back pain group (19 patients) and the non-residual back pain group (79 patients). The independent risk factors of residual back pain after PKP were screened and the scoring system was established by the multivariate logistic regression analysis. The performance of this scoring system was also prospectively validated using the clinical data of 45 patients with single-vertebral OVCF from January 2018 to December 2019.

**Results:**

The scoring system was consist of five clinical characteristics which were confirmed as significant predictors of residual back pain after PKP, namely, age ≥60 years (*P* = 0.021), fracture location = thoracic or lumbar (*P* = 0.002), fracture type = OF4 type (*P* = 0.018), segmental kyphosis ≥20° (*P* = 0.014), and bone cement volume <5 ml (*P* = 0.001). Patients in the residual back pain group showed a significant higher score than the non-residual back pain group (6.84 ± 1.71 vs. 2.66 ± 1.97, *t *= 8.499, *P *< 0.001), and the optimal cut-off value for the scoring system was 5 points. The sensitivity and specificity of the scoring system for predicting residual back pain after PKP were 84.21% and 87.34%, respectively, in derivation set and 78.57% and 83.87% in validation set.

**Conclusion:**

This novel scoring system showed satisfactory diagnostic efficacy in predicting residual back pain after PKP for single-vertebral OVCF. Patients with the score of 5–9 had a high risk of postoperative residual back pain, while the patients with score of 0–4 was low.

## Introduction

Osteoporosis is a systemic bone disease manifested by the decrease of bone density and quality, the destruction of bone micro-structure, and the increase of bone fragility, and often result in fractures (1). Osteoporotic vertebral compression fracture (OVCF) is the most common fracture type in osteoporosis patients. With the improvement of average life expectancy and the aggravation of population aging, the incidence of OVCF is increasing year by year (2). OVCF often leads to back pain, spinal kyphosis and even paralysis, thus seriously affect patients’ quality of life (3).

The treatment of OCVF included conservative treatment and surgical treatment. Conservative treatment mainly included bed rest, wearing waist brace, and analgesic drugs (4). However, patients' adherence to strict bed rest was not high and patients may suffer high risk of gastrointestinal bleeding due to the use of nonsteroidal anti-inflammatory drugs (NSAIDs) (5). Surgical treatment mainly included percutaneous vertebroplasty (PVP) and percutaneous kyphoplasty (PKP). Compared with PVP, PKP was reported with low risk of bone cement leakage and good ability of kyphosis correction, and thus is most commonly used at present (6, 7).

It was reported PKP can effectively relieve pain, and thus promote the early activities after surgery, shorten the time in bed, and reduce the risk of postoperative complications (8). Therefore, whether an OVCF patient can achieve effective pain relief after PKP was the focus of the attentions of both surgeons and patients (9). However, most of current studies focused on PKP-related complications, such as bone cement leakage and recurrent vertebral fractures (10, 11). Although the risk factors for non-relief of pain after PKP was reported (12), surgeons can hardly objectively and accurately predict the pain relief after PKP due to the so many reported risk factors and different effects of each risk factor on pain relief.

Therefore, we conducted this study to identify the clinical characteristics which can predict residual back pain and develop a novel scoring system to help spinal surgeon to predict residual back pain after PKP. We also validated the performance of this scoring system and confirmed its satisfactory ability in predicting residual back pain after PKP in OVCF.

## Materials and methods

This study was approved by the Ethics Committee of the Second People's Hospital of Chengdu and carried out in accordance with the Declaration of Helsinki. All of the participants provided their written informed consent to participate in this study. The work has been reported in line with the STARD criteria (13).

### Derivation of the scoring system

#### Patients selection

We retrospectively reviewed the medical records of hospitalized patients diagnosed of OVCF in our department from January 2015 to December 2017 to form the derivation set.

Inclusion criteria: (1) Acute single-vertebral OVCF (high signal in lipid suppressor sequence on MRI imaging); (2) Bone mineral density (BMD) examination (dual-energy x-ray absorption) confirmed osteoporosis (T score ≤−2.5); (3) The fracture type was OF2, OF3, or OF4 according to the Classification of Osteoporotic Thoracolumbar recommended by the Spine Section of the German Society for Orthopaedics and Trauma (DGOU) (14).

Exclusion Criteria: (1) Previous history of spinal surgery; (2) Long-term use of analgesics before hospitalization, such as NSAIDs, opioids; (3) Pathologic vertebral fracture caused by tumor, infection; (4) Less than 12-month follow-up or incomplete medical record data.

According to the patients selection criteria, a total of 98 patients were finally included in the derivation set with 29 males and 69 females. The average age of the included patients was 64.72 ± 7.89 years and the average disease course was 3.01 ± 1.45 months. The detailed clinical characteristics of the included patients were shown in Table 1.

**Table 1:** Clinical characteristics of the included patients.

Characteristic	Value
Number of patients (*n*)	98
Age (year, mean ± SD)	64.72 ± 7.89
Gender (*n*, %)	
Male	29 (29.59%)
Female	69 (70.41%)
Course of disease (month, mean ± SD)	3.01 ± 1.45
Fracture location (*n*, %)	
Thoracic vertebrae (T_4_–T_9_)	7 (7.14%)
Thoracolumbar vertebrae (T_10_–L_2_)	77 (78.57%)
Lumbar vertebrae (L_3_–L_5_)	14 (14.29%)
Fracture type (*n*, %)	
OF2/OF3 type	41 (41.84%)
OF4 type	57 (58.16%)
Segmental kyphosis (*n*, %)	
<20°	60 (61.22%)
≥20°	38 (38.78%)
Bone cement volume (*n*, %)	
<5 ml	44 (44.90%)
≥5 ml	54 (55.10%)
VAS (score, mean ± SD)	
Preoperative	8.45 ± 0.89
1 month postoperative	4.56 ± 0.76
6 months postoperative	5.18 ± 0.74

#### Data collection

Based on previous studies and our experience, we included the following predictors for residual back pain after PKP. In addition, postoperative VAS scores at different follow-up time were also recorded.

(1) Patient related data: (a) Age of patient: age ≥60 years or <60 years; (b) Gender of patient: male or female; (c) Disease course: ≥6 weeks or <6 weeks.
(2) Preoperative imaging data: (a) Fracture location: thoracic vertebrae (T_4_–T_9_), thoracolumbar vertebrae (T_10_–L_2_), or lumbar vertebrae (L_3_–L_5_); (b) Fracture type: OF3 type, OF3 type, or OF4 type; (c) Segmental kyphosis: the kyphosis angle was defined as the angle between the superior and inferior endplates of the fractured vertebra, kyphosis angle <20° or ≥20°.
(3) Surgery related data: bone cement volume <5 ml or ≥5 ml.
(4) Follow-up outcomes: VAS score at preoperatively, 1 month postoperatively, and 6 months postoperatively. Postoperative residual back pain was defined as the VAS score at 6 months postoperatively was more than four or the patients still need analgesic medication to contribute a good sleep.

#### Development of the scoring system

Firstly, the effects of these clinical characteristics, including age, gender, course of disease, fracture location, fracture type, segmental kyphosis, and bone cement volume, on postoperative pain (at 1 month and 6 months postoperatively) were all analyzed respectively. Secondly, all the included patients were divided into two groups, namely, non-residual back pain group and residual back pain group according to the 6-month postoperative follow-up outcomes. Next, multivariate logistic regression analysis was performed. According to the results of multivariate logistic regression analysis, the indexes with *P *< 0.05 were considered the final predictors for postoperative residual back pain and, thus, determined as the items of the scoring system. Then, we established the weighted score of each item based on the relative size of Odds Ratio (OR) according to the method reported by previous research (15). Finally, we identified the appropriate cut-off points for the scoring system using ROC curves corresponding to the point on the curve nearest the upper left corner of the ROC graph.

### Validation of the scoring system

From January 2018 to December 2019, we prospectively included patients to validate the accuracy of the scoring system. The following criteria were used to select patients to form the validation set. Inclusion criteria: (1) MRI suggested acute single-vertebral OVCF; (3) BMD examination showed T score ≤−2.5; (4) OF2, OF3, or OF4 type fracture. Exclusion Criteria: Exclusion Criteria: (1) Previous history of spinal surgery; (2) Long-term use of analgesics before hospitalization; (3) Pathologic vertebral fracture caused by tumor, infection.

Patients signed informed consent and then underwent PKP surgery. Before discharge, surgeon predicted whether the patient will suffer from residual back pain at 6 months postoperatively according to the scoring system (predictive outcome). At 6 months after surgery, the patient will be assessed whether they truly develop residual back pain (true outcome). The accuracy of the scoring system was evaluated by comparing the consistency between the predictive outcome and the true outcome.

### Statistical analysis

The effects of clinical characteristics on postoperative back pain at different follow-up point were analyzed by independent-samples *t-*test. The significant predictors of residual back pain at 6 months postoperatively were evaluated by multivariate logistic regression analysis. The items of the scoring system were determined by multivariate logistic regression, and the weighted score of each item was based on the relative size of the OR. The optimal cut-off point was made by using ROC curves. *P* < 0.05 was set of statistical significance. The SPSS version 10.0 software was used for statistical analysis.

## Results

### Derivation of the scoring system

**Figure 1:**
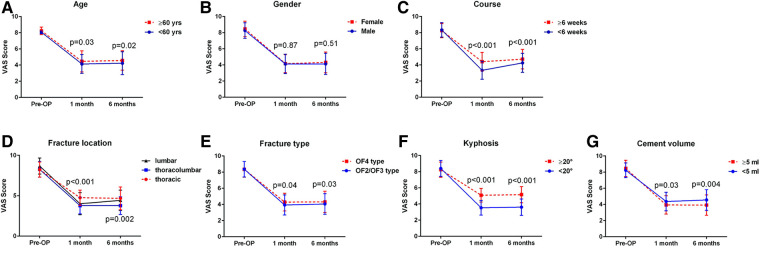
The effects of clinical characteristics on postoperative back pain at different follow-up point. (**A**), age; (**B**), gender; (**C**), course of disease; (**D**), fracture location; (**E**), fracture type; (**F**), segmental kyphosis; (**G**), cement volume.

It was showed that age ≥60 years (*P* = 0.03 and *P* = 0.02, respectively), course of disease ≥6 weeks (*P* < 0.001 and *P* < 0.001, respectively), fracture location = thoracic or lumbar (*P* < 0.001 and *P* = 0.002, respectively), fracture type = OF4 type (*P* = 0.04 and *P* = 0.03, respectively), segmental kyphosis ≥20° (*P* < 0.001 and *P* < 0.001, respectively), and bone cement volume <5 ml (*P* = 0.03 and *P* = 0.004, respectively), all had negative effects on postoperative back pain at both 1 month and 6 months postoperatively (Figure 1).

According to the back pain at 6 months postoperatively, 19 patients suffered residual back pain while 79 patients did not, and the incidence of postoperative residual back pain was 19.39%. Multivariate logistic regression analysis showed five clinical characteristics, namely, age ≥60 years (*P* = 0.021), fracture location = thoracic or lumbar (*P* = 0.002), fracture type = OF4 type (*P* = 0.018), segmental kyphosis ≥20° (*P* = 0.014), and bone cement volume <5 ml (*P* = 0.001) were significant predictors of postoperative residual back pain (Table 2).

**Table 2:** Multivariate logistic regression analysis of the risk factors of residual back pain after PKP.

Factors	Regression coefficient (*β*)	OR	*P*-value
Age ≥60 years	0.590	1.804	0.021
Fracture location = thoracic or lumbar	0.626	1.870	0.002
Fracture type = OF4 type	0.149	1.161	0.018
Segmental kyphosis ≥20°	0.720	2.054	0.014
Bone cement volume <5 ml	0.881	2.413	0.001

We developed a scoring system based on these five clinical characteristics that were confirmed significant predictors of postoperative residual back pain. The variables were given the weighted scores according to the relative value of the OR in multivariate logistic regression analysis: age ≥60 years, fracture location = thoracic or lumbar, fracture type = OF4 type, segmental kyphosis ≥20°, and bone cement volume <5 ml were weighted as 2 points, 2 points, 1 point, 2 points, and 2 points, respectively. The score was then calculated by determining the total number of points, ranging from 0 to 9 (Table 3).

**Table 3:** The scoring system for predicting residual back pain after PKP.

Variables	Score
Age	
≥60 years	2
<60 years	0
Fracture location	
Thoracic or lumbar vertebrae	2
Thoracolumbar vertebrae	0
Fracture type	
OF4 type	1
OF2 or OF3 type	0
Segmental kyphosis	
≥20°	2
<20°	0
Bone cement volume	
<5 ml	2
≥5 ml	0

**Figure 2:**
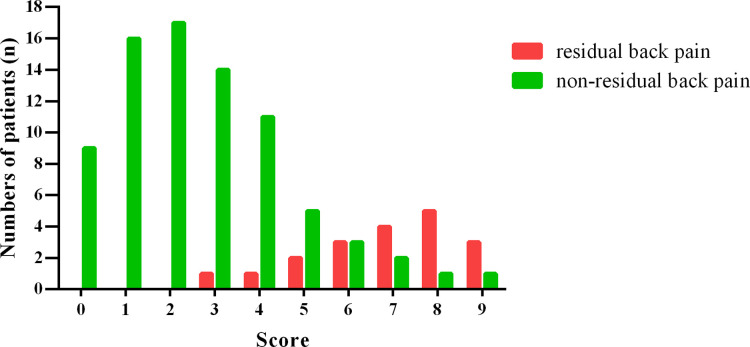
Histogram distribution of residual back pain group and non-residual back pain group for each score of the scoring system.

**Figure 3:**
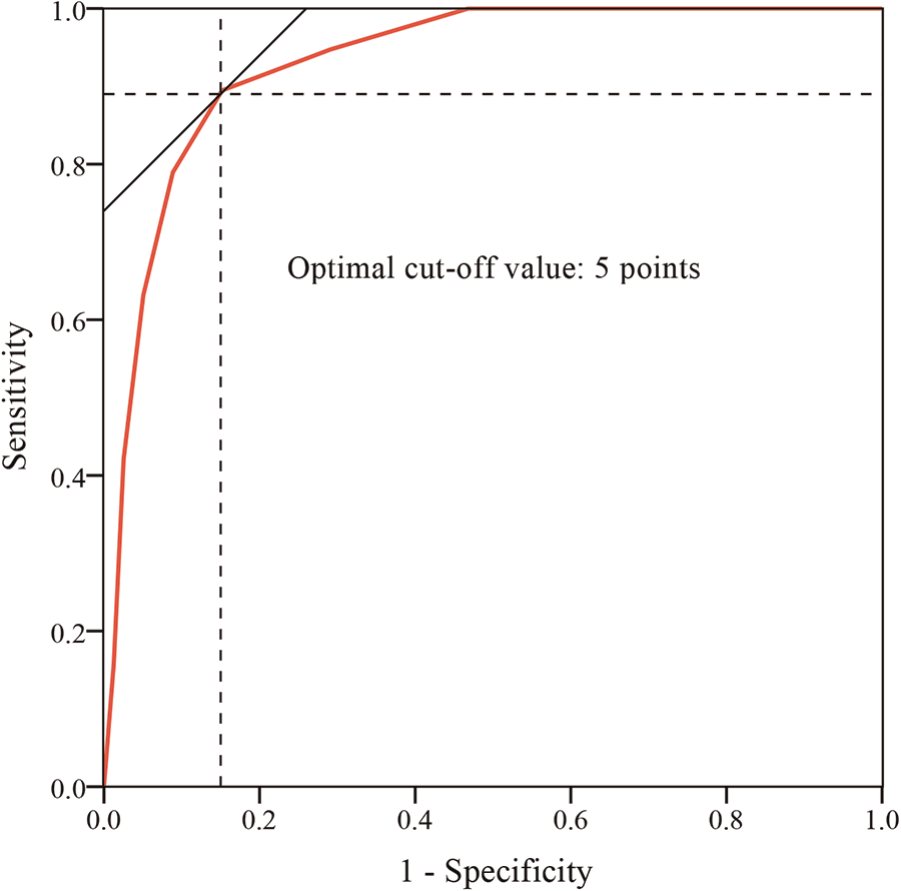
ROC curve analysis of the scoring system. The optimal cut-off point based on the ROC curve analysis of scores was 5 points.

A histogram distribution of the score values was shown in Figure 2. Remarkably, residual back pain group showed a significant higher score than non-residual back pain group (6.84 ± 1.71 vs. 2.66 ± 1.97, *t *= 8.499, *P *< 0.001). The optimal cut-off value of the predictive scoring system was 5 points, and the area under curve (AUC) was 0.931 (95% CI, 0.876–0.985, *P* < 0.001) (Figure 3).

### Validation of the scoring system

Finally, a total of 45 patients were prospectively included in the validation set, including 14 cases in residual back pain group group and 31 cases in non-residual back pain group according to the 6-month postoperative follow-up outcomes. Comparison of the performance of the score system on derivation set and validation set was shown in Table 4. Based on the cut-off value of 5 points, the sensitivity and specificity of the scoring system for predicting postoperative residual back pain were 84.21% and 87.34%, respectively, in derivation set and 78.57% and 83.87% in validation set.

**Table 4:** Comparison of performance of the scoring system on derivation set and validation set.

		Predictive outcome					
		Derivation set			Validation set		
		Residual back pain (score ≥5)	Non-residual back pain (score ≤4)	Total	Residual back pain (score ≥5)	Non-residual back pain (score ≤4)	Total
True outcome	residual back pain	16	3	19	11	3	14
	non-residual back pain	10	69	79	5	26	31
	Total	26	72	98	16	29	45
Sensitivity (%)		84.21			78.57		
Specificity (%)		87.34			83.87		

## Discussion

In this study, we first evaluated the clinical efficacy of PKP for OVCF. During the 6-month follow-up, we found that PKP was an effective procedure, with significant short-term relief of patients' pain symptoms. Although the VAS score of at 6 months postoperativbely was higher than that at 1 month postoperatively, the difference was not statistically significant, and the VAS score at 6 months postoperativbely was significantly lower than that before surgery, which further confirmed the efficacy of PKP for OVCF.

Moreover, we further analyzed the predictors of postoperative residual back pain. We found that the postoperative residual back pain 6 months after PKP was more obvious in OVCF patients aged ≥60 years than in patients aged <60 years. There might be the following reasons: (a) the degree of osteoporosis was more serious in the elderly (16); (b) the elderly had poor tolerance to surgical trauma and thus had slow postoperative recovery, long hospital stay and high risk of postoperative complications (17). This result was consistent with Yimin et al. who suggested that younger age was favorable factor for the prognosis of PKP (18). Diel et al. analyzed the prognostic factors of 1408 patients with vertebral fractures (including traumatic, pathological and osteoporotic) after PKP, and found that male patients had a higher risk of re-fracture after PKP (19). However, our present study found no significant difference in VAS scores between male and female OVCF patients. This may be because Diel et al. included vertebral fractures caused by multiple causes in their study, while our study only included vertebral fractures caused by osteoporosis. Yimin et al. suggested that PKP treatment within the first 6 weeks after fracture can achieve good pain relief (18). Our study found no significant relationship between course of disease and pain relief. This may be because Yimin et al.’s conclusions were drawn through literature review, and there may be publication bias.

In addition, the results of this study showed that there were significant differences in pain relief in different types of OVCF. First of all, patients with OF2 or OF3 type fracture had more obvious pain relief, while patients with OF4 type fracture had higher VAS scores after PKP. Secondly, we also found that OVCF patients with kyphosis had higher postoperative VAS scores and less pain relief. We speculate that this may be related to the severity of vertebral fracture (20), namely, burst fracture (or vertebral posterior margin fracture) and combined kyphosis suggested severe injury violence and vertebral fracture. In addition, we found that patients with thoracolumbar vertebral fracture had more obvious pain relief after PKP, while patients with thoracic or lumbar vertebral fracture had higher postoperative VAS scores, which may be related to higher anatomical stability of thoracic vertebral segments (21). By analyzing 27 patients with malignant vertebral fractures, Papanastassiou et al. also found that thoracic vertebral fractures had poor pain relief after PKP treatment (22). DGOU divided OVCF into 5 types (OF1–5) according to the injury of the posterior wall of the fracture vertebra. It was found that compared with OF4 and 5 types fractures, OF1–3 types fractures had better pain relief after PVP (23, 24), which was consistent with the findings of our present study. Therefore, we speculated that for patients with severe vertebral fractures (such as complete burst fractures), the addition of bone cement-assisted fixation on the basis of internal fixation may achieve better pain relief (25).

Surgical factors are important factors affecting the postoperative pain. Röder et al. conducted a retrospective analysis of 276 patients with single-segment vertebral fractures, and found that bone cement volume was an important factor influencing pain relief after PKP (OR = 0.36), meanwhile, they suggested that bone cement volume in PKP should be >4.5 ml in order to better relieve the pain (26). The above conclusions were consistent with our present study, which showed that bone cement volume <5 ml was a risk factor of residual back pain after PKP (OR = 2.412). Several studies suggested that there was a dose-effect relationship between pain relief and the bone cement volume, however, several studies showed a wireless relationship between the pain relief and the bone cement volume (27, 28). Al-ali et al. believed that neither the amount of bone cement and the leakage of bone cement to intervertebral disc had correlation with pain relief after PKP (29). The main reasons were as follows (30, 31): (a) different definitions of pain relief in these studies; (b) different sites of vertebral fractures were included in each study; (c) the evaluation indexes of bone cement volume are different. Therefore, in our opinion, when PKP was used for OVCF, the optimal bone cement volume should be individual (32). When the bone cement volume is low, the pain relief may not be satisfactory, while when the bone cement volume is too high, the risk of complications may be increased, such as bone cement leakage and adjacent vertebral fractures.

There are some limitations of our study. First, it was a retrospective study. Second, the number of patients included in this study was small and the postoperative follow-up time was short. Third, this study did not evaluate other factors that might affect postoperative pain relief, such as comorbidities.

In summary, our study suggested that PKP can significantly relieve pain in OVCF patients. Age ≥60 years, fracture location = thoracic or lumbar, fracture type = OF4 type, segmental kyphosis ≥20°, and bone cement volume <5 ml were the predictors for residual back pain after PKP. Due to the limitations of the study, further studies are needed to confirm the above conclusion.

## Data Availability

The raw data supporting the conclusions of this article will be made available by the authors, without undue reservation.
